# Acute Joint Blockage due to Abrasion-Related Dislocation of a Silastic Radial Head Prosthesis: A Histological Examination after 14 Years of Durability

**DOI:** 10.1155/2020/8840087

**Published:** 2020-08-10

**Authors:** Heinz-Lothar Meyer, Christina Polan, Anke Bernstein, Benedikt Abel, Manuel Burggraf, Marcel Dudda, Max Daniel Kauther

**Affiliations:** ^1^Department for Trauma, Hand and Reconstructive Surgery, University Hospital Essen Germany, Hufelandstraße 55, 45147 Essen, Germany; ^2^G.E.R.N. Laboratory for Tissue Replacement, Regeneration and Neogenesis, University Hospital Freiburg, Engesserstraße 4, 79108 Freiburg, Germany

## Abstract

The implantation of a radial head prosthesis can take place as a therapeutic option after radial head fracture. There are various implants for this purpose. Many studies and case reports about silastic radial head prosthesis implantation describe foreign body reactions with accompanying synovitis and poor functional results. A few studies have investigated the reason for the material failure and the accompanying synovitis. The case report presented shows an unusually long durability of an in situ 14-year silastic radial head prosthesis. 14 years after implantation, a previously full-time working and healthy patient presented himself with a dislocation of the silastic radial head prosthesis and atraumatic joint blockage of the right elbow triggered by a negligible movement. The prosthesis was removed surgically. We found a macroscopic foreign body reaction intraoperatively. In a histopathological examination, with hematoxylin and eosin staining (HE) in 40x and 100x magnification, we have seen an aseptic inflammatory response to foreign bodies with activated epithelial cells and multinucleated giant cells with intracytoplasmic foreign material. Due to these problems, the silastic radial head prosthesis is no longer used today. However, there are still patients with the implanted silastic radial head prosthesis, which should therefore be checked regularly. A metal prosthesis also does not seem to be an optimal alternative due to cartilage wear and loss of ROM. The choice of prosthesis material should be selected carefully and patient-specific in radial head prosthetics according of the results presented.

## 1. Introduction

Radial head fractures account for one-fifth of all injuries in the area of the elbow. Therapy can include osteosynthetic treatment, resection of the entire radial head, or implantation of a radial head prosthesis [1–3]. There are various implants for the latter [4]. A study by Maghen et al. showed no complications in a follow-up period of an average of five years after implantation of silastic radial head prostheses, with simultaneous surgical restoration of the ligamentous apparatus of the elbow [5]. A systematic review and meta-analysis from Kachooei et al. shows that the highest incidence of removal/revision occurred within 2 years after implantation of a radial head prosthesis. Most radial head prostheses have an acceptable and comparable midterm longevity in incidence of removal and revision. However, the long-term results are still unclear in this study [6]. Hence, many studies and case reports after implantation of silastic prostheses describe foreign body reactions with accompanying synovitis and poor functional results [7, 8]. The study by Berger et al. shows that on average, material failure occurs after five years [9]. A few studies have investigated the reason for the material failure and the accompanying synovitis. They showed inflammatory arthritis, reactive synovitis, and dislocation of the prosthesis after radial head replacement with a silastic head prosthesis [10, 11]. Due to these problems, the silastic radial head prosthesis is no longer used today. However, there are still patients with the implanted silastic radial head prosthesis, which should be checked therefore regularly [6]. Histopathological and surface investigations on the mechanism failure in the implantation of silastic radial head prostheses are presented. No histological studies to explain the reason for implant failure are described in the literature.

## 2. Case Presentation

**Figure 1:**
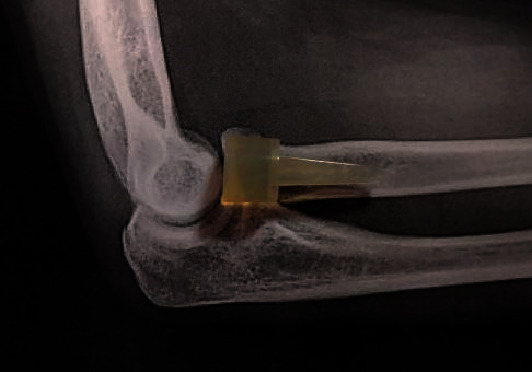
Schematic representation of the silastic radial head prosthesis in a lateral X-ray of an elbow.

**Figure 2:**
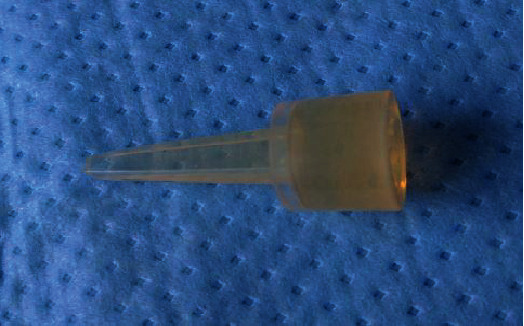
New silastic radial head prosthesis.

We report on a 39-year-old male patient who suffered a Mason IV radial head fracture due to an occupational accident 14 years ago. Initially, a silastic radial head prosthesis (size 3, Wright Medical Technology Inc.) was implanted as radial head replacement (Figures 1 and 2).

In the follow-ups at one, three, and ten years after implantation, an equal range of motion (ROM) for both elbows of Flex/Ex 140/0/0° and Sup/Pro 90/0/90° was shown. There was no complaint about arm strength restrictions. The patient worked as a cook with full work ability and no complaints. The examination was carried out by a senior attending physician.

**Figure 3:**
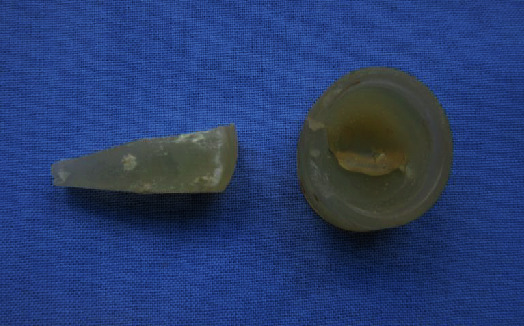
Silastic radial head prosthesis after 14 years of durability after operative removal.

**Figure 4:**
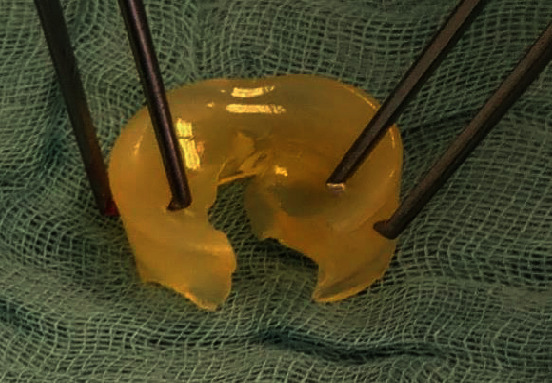
Crack in the explanted silastic radial head prosthesis after 14 years of durability.

**Figure 5:**
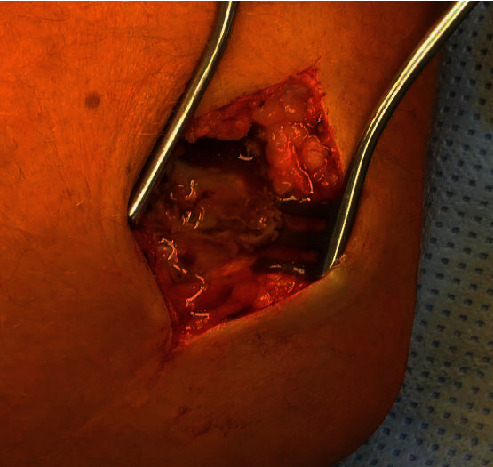
Macroscopic intraoperative finding on the right elbow: foreign body reaction.

The patient presented to our emergency room 14 years after the implantation of the silastic radial head prosthesis with pain in his right elbow. The acute symptoms with painful joint blockage occurred after a negligible movement of the elbow by driving a car. On admission, there were no irritated scars and no pressure pain on the right elbow. The ROM was raised by a senior attending physician for Flex/Ext 100/30/0°. The patient's examination for supination and pronation demonstrates a final restriction of movement. Peripheral blood circulation, motor function, and sensitivity were intact. The radiological X-ray examination showed a dislocated silastic prosthesis in the right ventral elbow without signs of macroscopic osteolysis. There were no abnormalities in the laboratory (leukocytes 8.19/nl and CRP < 0.5 mg/dl). Intraoperatively, there was a longer separation of the spacer and shaft parts and acute tears in the head part, probably due to the dislocation mechanism (Figures 3 and 4). An intraoperative swab showed no evidence of septic loosening. The prosthetic head dislocated into the ventral elbow joint capsule, leading to joint blockage (Figure 5).

**Figure 6:**
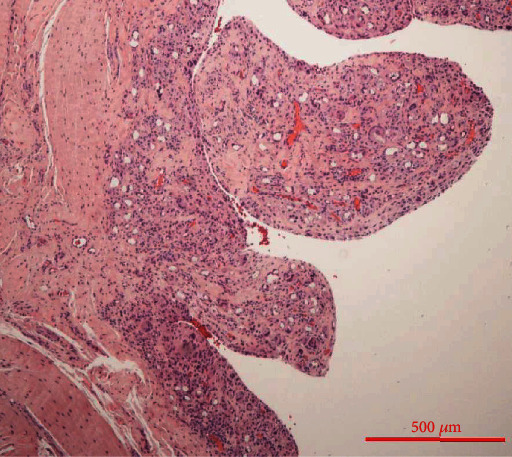
Histopathology. Hematoxylin and eosin staining (HE), 40x maginification, aseptic inflammatory response to foreign bodies.

**Figure 7:**
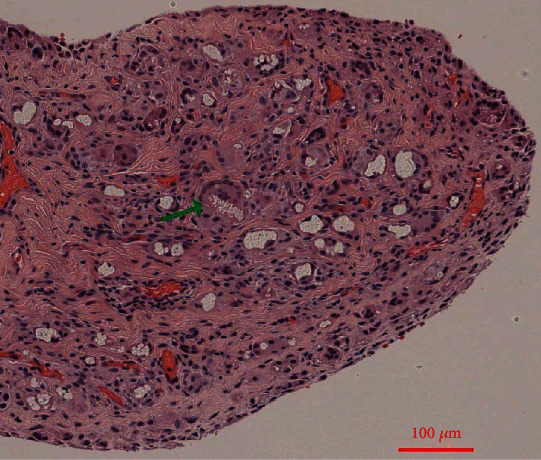
Histopathology. HE staining, 100x maginification, aseptic inflammatory response to foreign bodies with activated epithelial cells and multinucleated giant cells with intracytoplasmic foreign material (green arrow).

**Figure 8:**
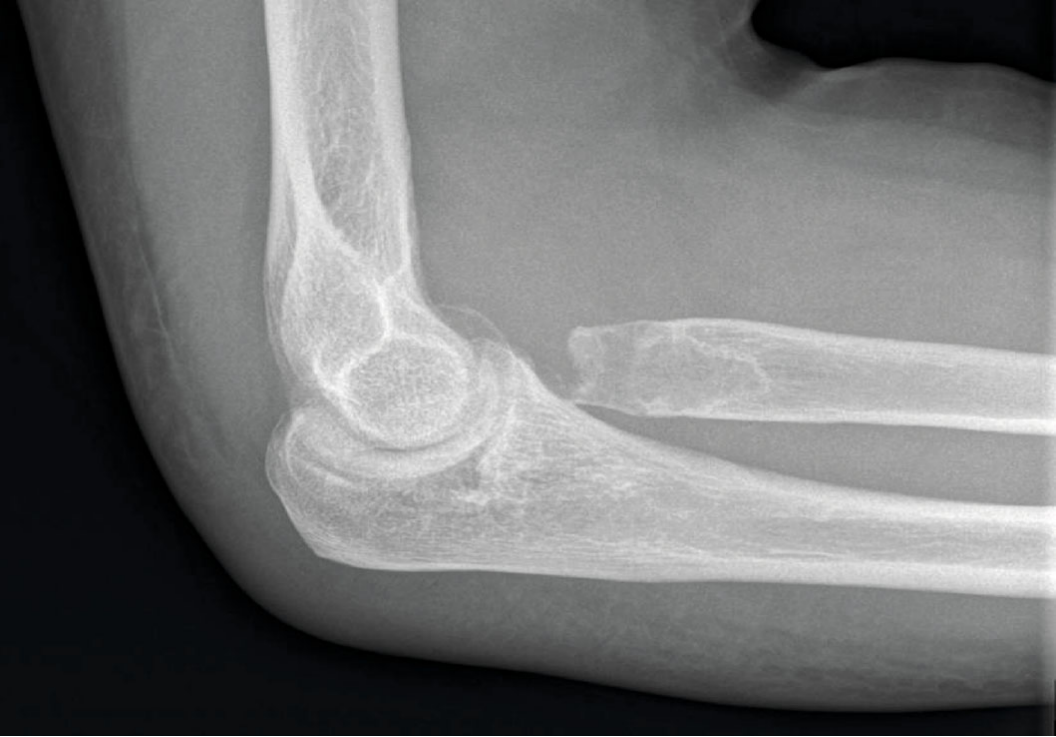
Lateral X-ray of the elbow after removal of the silastic radial head prosthesis.

Histopathologically, there was an aseptic inflammatory response to foreign bodies with activated epithelial cells and multinucleated giant cells with intracytoplasmic foreign material (Figures 6 and 7). The prosthesis was removed intraoperatively (Figure 8). After completing the therapy, the patient was satisfied and free of symptoms, with a free ROM.

## 3. Discussion

The presented case shows an atypically long durability of a silastic radial head prosthesis in situ. As previously described, 14 years after implantation, we can also demonstrate macroscopic and microscopic-histological foreign body reactions (Figures 3, 6, and 7) [5, 7, 8, 12]. Many studies and case reports after implantation of a silastic prosthesis describe foreign body reactions with accompanying synovitis and poor functional results [7, 8]. Inflammatory arthritis, reactive synovitis, and dislocation of the prosthesis after radial head replacement with a silastic head prosthesis are also described in the literature [10, 11, 13]. After five years, an average of a material failure is often described [8]. In this case, we also see an aseptic inflammatory reaction to foreign bodies with activated epithelial cells and multinucleated giant cells with intracytoplasmic foreign material (Figures 6 and 7).

Just like in previous studies, we saw intraoperatively a longer separation of the spacer and shaft parts and acute tears in the head part, probably due to the dislocation mechanism (Figures 2–5) [10, 11].

However, material failure occurred not until 14 years after implantation. A study by Petitjean et al. showed that the implantation of a silastic prosthesis as a temporary spacer represents good functional results for an average of eight months before explantation became necessary [12]. The study also showed that it prevents synovitis due to abrasion and implant failure [12]. Implantation of a metal prostheses does also not appear to be an optimal alternative due to cartilage wear and loss of ROM over the course [5, 14, 15].

However, other studies by Carità et al. and Kachooei et al. show that radial head prostheses show good results in patients with complex fractures and poor prognosis of the radial head with a well-positioned and correctly selected prosthesis. This suggest that the use of prostheses, if well positioned and selected, is a valid solution in the treatment of complex fractures of the radial head with poor prognosis. Good results in the reduction of pain, recovery of movement, and improved quality of life were shown [4, 6]. This result is supposedly limited in time, as shown in our case report. In order to prove this significantly, a larger case series or study would have to be carried out.

The presented silastic radial head prosthesis (Wright Medical Technology Inc.) was taken off the market, because of the side effects described. In radial head prosthetics, the choice of prostheses material should be selected carefully and in a patient-specific manner in view of the results presented [13, 16, 17].
